# Fosfomycin-Induced Liver Injury: A Case Report

**DOI:** 10.7759/cureus.55508

**Published:** 2024-03-04

**Authors:** Rui Ribeiro, Judite Antas, Ana Pais Monteiro, José Magalhães, Diana Miranda, Célia Cruz

**Affiliations:** 1 Internal Medicine, Centro Hospitalar Universitário de Santo António, Porto, PRT

**Keywords:** idiosyncratic liver injury, hepatocellular liver injury, hepatotoxic drugs, drug-induced liver injury (dili), fosfomycin

## Abstract

Fosfomycin is an antibiotic frequently used to treat uncomplicated urinary tract infections. It is normally well-tolerated, but there are some reports of clinically relevant liver injury. We present the case of a 73-year-old female who presented with paucisymptomatic hepatocellular acute liver injury six days after taking fosfomycin. After ruling out viral, ischemic, and autoimmune hepatitis, as well as Wilson disease and biliary disorders, she was diagnosed with drug-induced liver injury (DILI) related to fosfomycin. The patient showed major improvement during the first week and the resolution of liver injury one month after onset. This case report aims to underscore the potential hepatotoxicity of fosfomycin.

## Introduction

Fosfomycin is a phosphoenolpyruvate analogue that is used as a broad-spectrum antibiotic [1]. It is commonly used in the treatment of uncomplicated urinary tract infections since it is effective against most common urinary pathogens [1]. In Portugal, it is one of the first-line treatment options to treat acute cystitis. Recently, due to the development of multidrug-resistant bacteria, fosfomycin has been used in hospitals to treat complicated infections [1].

In general, fosfomycin is well-tolerated with mild and self-limited gastrointestinal symptoms such as diarrhea, nausea, and abdominal pain being the most common adverse events [2]. Mild elevations of serum aminotransferase levels occur in a small percentage of patients taking oral fosfomycin [2].

Drug-induced liver injury (DILI) can be classified as intrinsic (dose-related with onset within a short time span) or idiosyncratic (not dose-related with a variable latency to onset of days to weeks) [3].

There is a small number of reports of idiosyncratic liver injury attributed to fosfomycin, and they are diverse when it comes to the route of administration, the pattern of liver injury, and time to resolution [4]. Nonetheless, the injury seems to be self-limited, and the time to onset is one week after exposition [4]. The mechanism of injury is unknown [4]. No fatal instances of acute hepatitis have been reported [4].

## Case presentation

A 73-year-old female with a medical history of hypertension and dyslipidemia presented to the emergency department with complaints of epigastric pain and dyspepsia for three days before admission. She denied other symptoms such as fever, nausea, vomiting, choluria, or acholia. She had been taking valsartan, amlodipine, and simvastatin for several years with a normal liver enzyme panel from three months prior to admission. Six days before admission, the patient took a single 3 g dose of fosfomycin and 200 mg of flavoxate three times a day for acute cystitis prescribed by her general practitioner. She denied taking any herbal or over-the-counter medications, mushroom consumption, and recent trips outside Portugal in the previous year. There was also no history of syncope in the days preceding admission.

On physical examination, vital signs and abdominal examination were normal, and there was no finding suggestive of encephalopathy.

The blood test summarized in Table 1 showed elevated levels of aminotransferases, gamma-glutamyl transferase, and alkaline phosphatase without leukocytosis or significative elevation of C-reactive protein.

**Table 1:** Laboratory results upon admission.

Laboratory finding	Admission	Reference range
Hemoglobin, g/dL	15.4	12-15
Leucocyte count, *×*10^3^/µL	8.48	4-11
Neutrophil count, *×*10^3^/µL	4.1	2-7.5
Platelet count, *×*10^3^/µL	309	150-400
Total bilirubin, mg/dL	1.69	0.2-1.1
Direct bilirubin, mg/dL	1.3	0.0-0.3
Alkaline phosphatase, IU/L	344	35-104
Gamma-glutamyl transferase, IU/L	450	6-39
Aspartate aminotransferase, IU/L	1236	10-30
Alanine aminotransferase, IU/L	1421	10-36
Albumin, g/dL	4.15	3.4-4.8
Prothrombin time, seconds	11.4	11.4
Creatinine, mg/dL	0.81	0.5-0.9
Lipase, IU/L	33	30-190
Amylase, IU/L	29	0-53
C-reactive protein, mg/L	5.13	0-5

Abdominal ultrasound showed the presence of gallstones in the gallbladder without pericholecystic fluid, sonographic Murphy sign, or the dilation of the intra- and extrahepatic bile ducts. Liver ultrasonography showed increased echogenicity, excluding focal lesions.

Given these results, cholecystitis and cholangitis were excluded. The patient was admitted for further investigation of acute liver injury.

Immunoglobulin M (IgM) anti-hepatitis A virus (HAV), hepatitis B surface antigen (HBsAg), IgM anti-hepatitis B core (HBc), anti-hepatitis C virus (HCV), anti-HIV, IgM anti-cytomegalovirus (CMV), and IgM anti-Epstein-Barr virus (EBV) antibodies were negative. Serum hepatitis E virus (HEV) RNA was untraceable. Immunoglobulin G antibody, antineutrophil cytoplasmic antibody (ANCA), anti-liver-kidney microsomal (LKM) antibody, anti-mitochondrial antibody (AMA), anti-smooth muscle antibody (ASMA), 24-hour urinary copper, and ceruloplasmin were within normal range.

**Figure 1:**
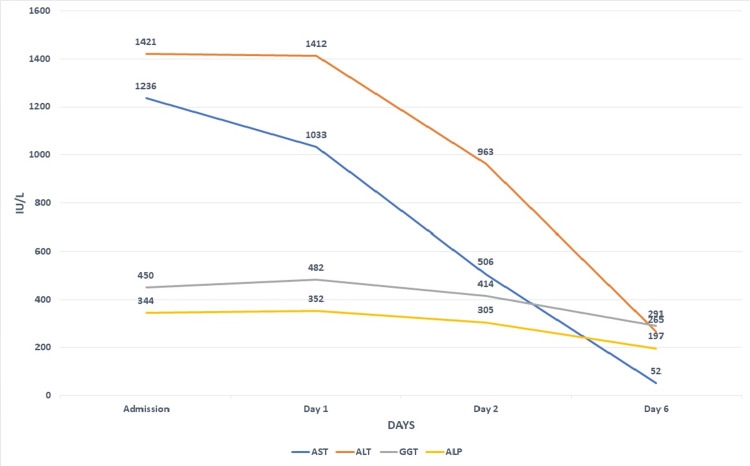
Trend of liver enzymes during hospital stay. AST, aspartate aminotransferase; ALT, alanine aminotransferase; GGT, gamma-glutamyl transferase; ALP, alkaline phosphatase

The patient remained asymptomatic during her hospital stay. No specific treatment was carried out. Aminotransferase levels improved continuously, and she was discharged seven days after admission; see Figure 1.

Given this improvement, it was decided not to proceed with a liver biopsy. The patient was reevaluated one month after discharge in the outpatient clinic with normalized transaminase levels. She was advised to avoid fosfomycin in the future.

## Discussion

Drug-induced liver injury is a challenging clinical condition because of the variety of drugs used in practice, the diversity of clinical and pathological phenotypes, and the absence of specific biomarkers [3]. The first step when dealing with a suspected case of DILI is to exclude signs of other organ involvement or hepatic failure that might indicate severe toxicity and a need for advanced supportive care. It is by nature an exclusion diagnosis; thus, it is crucial to search for the presence of viral infections, alcohol-related liver disease, hepatic ischemia, autoimmune hepatitis, Wilson disease, and biliary disorders [3]. The R factor is used to define the pattern of liver injury. It is equal to the patient’s alanine aminotransferase (ALT)/ALT upper limit of reference range divided by alkaline phosphatase (ALP)/ALP upper limit of reference range. An R value of more than 5 indicates a hepatocellular pattern of liver injury, whereas a value of less than 2 refers to a cholestatic-type pattern; a value between 2 and 5 represents a mixed pattern of DILI [5].

The updated Roussel Uclaf Causality Assessment Method (RUCAM) scale provides a more objective tool when assessing the possibility of DILI based on seven different domains: temporal relationship between exposure to the drug and liver injury, aminotransferase evolution, risk factors, concomitant drug use, the exclusion of alternative nondrug-related etiologies, evidence regarding DILI with that specific drug, and response to reexposure to the medication [6]. The total score ranges from -9 to 10, and it classifies each case as highly probable (≥9), probable (6-8), possible (3-5), unlikely (1-2), or excluded (≤0) [6].

The first-line management of DILI is to discontinue the implicated agent. In the vast majority of cases, a spontaneous recovery occurs, without the need for any specific treatment [3].

Our patient presented with acute liver injury and aminotransferase levels greater than 1000 IU/L, suggestive of toxin or drug-induced liver injury, acute ischemic liver injury, acute viral hepatitis, severe autoimmune hepatitis, or Wilson disease [7]. There was no history suggestive of a major hemodynamic occurrence prior to admission, so acute ischemic liver injury was excluded. As stated in the case presentation, acute hepatitis caused by viruses A, B, C, and E; HIV; EBV; and CMV were ruled out. Since there was no evidence of immunosuppression or mucocutaneous abnormalities, infection with herpes simplex virus and varicella zoster was deemed very unlikely. Immunological and cooper metabolism laboratory findings were not indicative of autoimmune hepatitis or Wilson disease.

There were two agents in an adequate time frame for acute DILI: flavoxate and fosfomycin. Flavoxate is a synthetic anticholinergic agent with no evidence of hepatotoxicity [8]. Fosfomycin is a rare cause of liver injury [2] presenting with mild aminotransferase elevations in 1%-2% of patients taking this drug.

The R factor of the case reported was 12, which is indicative of a hepatocellular pattern of liver injury. Based on the RUCAM scale, a score of 8 was obtained in our case, indicating that the association with fosfomycin was “probable.” A drug rechallenge would be important to establish evidence, but it was not considered for ethical reasons.

To the authors’ knowledge, there are four case descriptions of moderate or severe fosfomycin-induced liver injury in the available literature. Matsumori et al. [9] reported a case of a 30-year-old male who developed a mixed-pattern liver injury three days after taking oral fosfomycin. He developed features of liver failure (encephalopathy and coagulopathy), but there was no need for liver transplantation, and the normalization of liver enzymes was achieved four months after onset. Durupt et al. [10] published the case of a 30-year-old female with cystic fibrosis who developed liver injury four days after starting parenteral fosfomycin, with the resolution of liver injury one month after antibiotic discontinuation. This patient was rechallenged twice with liver enzymes increasing three days after fosfomycin administration and returned to normal levels within a week. The ALP value was not mentioned; the liver injury pattern was not classified. Wang et al. [11] described a series of 30 cases of DILI mentioning a 50-year-old male who presented with a mixed liver injury three days after exposure to fosfomycin with resolution within a week. The administration route was not mentioned. Ferreira et al. [12] reported a case of a 24-year-old female presenting with a hepatocellular liver injury one week after taking a single 3 g dose of oral fosfomycin. Resolution was noted three months after exposure.

Case descriptions of fosfomycin-induced liver injury are scarce, making it difficult to establish a pattern of clinical presentation. Nonetheless, the case presented in this article shares some features with all the previous reports, namely, the onset within one week of exposure and the self-limited clinical course without the need for liver transplantation. Hepatocellular was also one of the patterns previously described for this drug, and the time to resolution is consistent with some of the previously published cases. The authors think that these features strengthen the causal relationship established between the drug and liver damage.

Further case reports or even case series would be helpful to better understand fosfomycin-induced liver injury.

## Conclusions

Fosfomycin is a commonly used antibiotic and is usually well-tolerated without serious adverse effects, but it has been associated with DILI. In patients with unexplained liver injury, DILI should be promptly considered as the discontinuation of the drug is the mainstay of treatment. This case report highlights the need for awareness of fosfomycin-induced liver injury as a rare but potentially serious side effect. It also reinforces previous knowledge on the clinical presentation of this entity such as time to onset within a week of treatment and self-limited course.

Reports of rare drug adverse effects are important because they increase knowledge and lead to better-informed clinical decisions.
